# Establishment and validation of a dual qPCR method for the detection of carbapenem-resistant *Acinetobacter baumannii* in bloodstream infections

**DOI:** 10.3389/fcimb.2025.1490528

**Published:** 2025-02-26

**Authors:** Lin Yu, Xianglan Kou, Ze Liu, Chushi Guan, Baoqing Sun

**Affiliations:** ^1^ Department of Clinical Laboratory, Guangzhou Institute of Respiratory Health, State Key Laboratory of Respiratory Disease, National Center for Respiratory Medicine, National Clinical Research Center for Respiratory Disease, Guangzhou Laboratory, The First Affiliated Hospital, Guangzhou Medical University, Guangzhou, China; ^2^ KingMed School of Laboratory Medicine, Guangzhou Medical University, Guangzhou, Guizhou, China

**Keywords:** bloodstream infections, carbapenemase resistance, *Acinetobacter baumannii*, qPCR, methodology

## Abstract

**Objective:**

Bloodstream infections(BSIs) caused by carbapenem-resistant *Acinetobacter baumannii* (CRAB) have a high mortality rate due to the high levels of drug resistance. There is an urgent need to establish a sensitive and accurate detection method to rapidly detect CRAB in BSIs.

**Methods:**

A new method was developed based on fluorescence quantitative PCR (qPCR) targeting the specific region of 16sRNA and OXA-23 gene from CRAB. The parameters were evaluated and optimized. This qPCR method was further applied in the detection of AB from 30 clinical specimens.

**Results:**

The qPCR method established in this study showed high specificity. The method successfully differentiated *Acinetobacter baumannii*(A. baumanii) from 26 other common pathogens in BSIs and identify the carbapenem resistance gene. The qPCR method shows a limit of detection (LOD) of 3×10^-3^ ng/μL, and displays good linear relationship between 16sRNA and OXA-23 and excellent repeatability (CV ≤2%). The results for the detection of 30 clinical specimens using this new qPCR method are in complete agreement with those using blood culture and drug susceptibility test.

**Conclusion:**

The qPCR method established in this study has strong specificity, wide linear range, good repeatability, and a lower LOD than PCR (Non-fluorescence quantification). The method provides new technical support for the early clinical diagnosis of CRAB in BSIs.

## 1 Introduction

Bloodstream infections (BSIs) is a severe, life-threatening systemic infectious disease (Xu et al., 2023b). In BSIs, especially infections caused by multidrug-resistant bacteria such as carbapenem resistant gram-negative bacilli, the mortality rate is high (Falcone et al., 2023). *Acinetobacter baumannii*(A. baumanii) is a gram-negative bacillus that causes pathogenic conditions, and it is one of the common pathogenic bacteria that cause nosocomial infections (Xu et al., 2023a). The key to the treatment of BSIs is the timely and accurate diagnosis of pathogenic bacteria and their drug resistance.

Carbapenem antibiotics, including imipenem and meropenem, are regarded as the last line of defense for the treatment of AB infection (Ranjbar and Alam, 2023). Once the resistance occurs, it will endanger life (Murray et al., 2022). According to the China Antimicrobial Surveillance Network, the resistance rates of A. baumanii to meropenem and imipenem were 71.4% and 68.6% in 2016 (Hu et al., 2017). Although efforts have been made to standardize the use of antibiotics in recent years, the resistance rates of the two antibiotics were still as high as 73.7% and 73.4%. in 2023, showing a basic year-on-year increasing trend (Hu et al., 2022).

OXA type carbapenemase is the most important cause of carbapenem resistance in A. baumanii worldwide. It belongs to class D enzymes in the Ambler molecular classification. OXA-23 is the most common genes (Ramirez et al., 2020; Tian et al., 2024). Previous studies have shown that OXA-23 is detectable in carbapenem-resistant *Acinetobacter baumannii* (CRAB), but it is absent in carbapenem-sensitive *Acinetobacter baumannii*(CSAB) (Zhang et al., 2022). This detection rate is consistent with other relevant studies in China (Shi et al., 2021; Guo et al., 2023). This explains that the OXA-23 genotype is closely related to the carbapenem-resistant phenotype (Koirala et al., 2020; Gupta et al., 2022).

In order to help clinical timely diagnosis and precise treatment, this study aims to design a 16sRNA/OXA-23 dual qPCR method to detect CRAB in BSIs. Utilizing the specificity of the variable region of A. baumanii’s 16sRNA, we can distinguish A. baumanii from other pathogenic microorganisms; Simultaneously, OXA-23 is a dominant gene for carbapenem resistance in A. baumanii. So the primers and probes were designed and optimized according to them (Ibrahim et al., 2021).

## 2 Materials and methods

### 2.1 Experimental materials

#### 2.1.1 Sample source

The samples used in the establishment of the methodology of were all isolated from the microbiology laboratory of the First Affiliated Hospital of Guangzhou Medical University from July 2022 to April 2024. Pathogenic microorganisms in whole blood samples are isolated and identified according to the National Clinical Laboratory Operation Procedures (Fourth Edition) (Hong et al., 2015).

#### 2.1.2 Instruments and reagents

Fluorescence qPCR instrument (Bio-rad), micropipette (Eppendorf), high-speed centrifuge (Eppendorf), vortex mixer (Hemendirin Bell), LX-200B compound rotor centrifuge (Hemendrin, Bell), biological safety cabinet (Sailen Instrument), Probe qPCR Mix (Takara), primers and probes (Sangon Bioengineering Co., Ltd.), Colombian blood plates (Antu Biologics), QIAamp DNA purification mini kit (QIAGEN GmbH).

### 2.2 Methods

#### 2.2.1 Collection and preservation of strains

Collect A. baumanii isolated and cultured from positive clinical blood culture and other bacteria and fungi with positive blood culture in clinical practice, and collect the corresponding colonies on the blood agar plate, chocolate plate or fungus selective culture plate identified by the mass spectrometer into the preservation tube, and keep the number in the -80°C refrigerator for later use.

#### 2.2.2 Extraction of bacterial DNA

The bacterial DNA of the bacteria specimens collected in the clinic were extracted by column extraction, the strains were thawed, the strains were separated by partitioning on the blood agar plate, and the incubator was incubated at 37°C for 8 hours to 24 hours, the DNA was extracted using the bacterial extraction kit, the DNA concentration and purity were detected with NanoDrop2000, and finally stored in the -20°C refrigerator. The bacterial DNA from Venous blood samples were extracted by QIAamp DNA Mini Kit(50) reagents (QIAGEN).

DNA concentration and purity were measured and recorded, and stored in -20°C freezer. 3. Synthesis of primers and probes: by searching the gene sequences of 16sRNA and OXA-23 in the NCBI database, design and optimize the relevant primer and probe sequences and primer sequences (**Supplementary Table S1**).

#### 2.2.3 Dual qPCR reaction system construction and condition optimization

##### 2.2.3.1 Dual qPCR system construction

Design a dual qPCR reaction system with a total volume of 20 μl, in which primers and probes are added at a concentration of 10 μM. This included 10 μl of qPCR mix, 1 μl of each of the forward and reverse primers for OXA-23/16sRNA, 0.5μl of the OXA-23/16sRNA probe and 2 μl of target DNA, with the volume made up to 20 μl with distilled water. According to the Probe qPCR Mix reagent instructions, the PCR amplification procedure was determined as follows: pre-denaturation at 95°C for 30 s; Denaturation 95°C, 5s; annealing 60°C, 30 s; a total of 39 cycles.

Sterile water was set as the negative control, and different CRAB and CSAB were used to verify the feasibility of primers, probes and dual qPCR reaction with different CRAB and CSAB.

##### 2.2.3.2 Optimization of dual qPCR primer concentration

The primer concentration is 500 nM and the probe concentration is 250 nM when the system is constructed. The optimal primer concentration was determined by setting the primer concentration of 300 nM, 400 nM, 500 nM, and 600 nM according to the common primer concentration, and the probe concentration was adjusted according to half of the primer concentration. The optimal primer concentration is determined based on the fluorescence intensity background and the size of the Ct value.

##### 2.2.3.3 Optimization of dual qPCR 16sRNA/OXA-23 primer probe concentration ratio

According to the primer optimization results, the 16sRNA/OXA-23 primer concentration was set to 300 nM:500 nM, 400 nM:500 nM, 500 nM:500 nM, 500 nM:600 nM, 400 nM:600 nM. The probe concentration is adjusted by half the amount of primer concentration. The optimal primer concentration is determined based on the size of the Ct value.

##### 2.2.3.4 Dual qPCR annealing temperature optimization

After determining the optimal primer concentration and primer probe concentration ratio, set the gradient annealing temperature to optimize the annealing temperature. In this experiment, the intermediate temperature was set at about 60°C, and finally 65°C, 64.5°C, 63.4°C, 61.9°C, 60°C, 58.4°C, 57.3°C, and 56.7°C were used as the degradation temperatures to be optimized in this experiment.

#### 2.2.4 Double qPCR specificity verification

22 common bacteria, 3 fungi and 1 virus were selected for specific verification of common bloodstream infection pathogens in the hospital. Sterile water was used as a negative control and CRAB was used as a positive control. The associated pathogens are as follows:

Bacteria: Escherichia coli, Klebsiella pneumoniae, Staphylococcus epidermidis, Staphylococcus aureus, Staphylococcus capitis, Pseudomonas aeruginosa, Enterococcus faecium, Enterococcus faecalis, Staphylococcus hominis, Staphylococcus haemolyticus, Enterobacter cloacae, Proteus mirabilis, Propionibacterium acnes, Stenotrophomonas maltophila, Streptococcus pneumoniae, Staphylococcus lugdunensis, Klebsiella oxytoca, Corynebacterium striatum, Serratia marcescens, Staphylococcus warneri, Morganella morganii; Viruses: Hepatitis B virus; fungi: Candida albicans, Candida tropicalis, Candida glabrata.

#### 2.2.5 Dual qPCR limit of detectionand linearity experiment

The A. baumanii DNA was diluted to 3×10^2^, 3×10^1^, 3×10^0^, 3×10^-1^, 3×10^-2^, 3×10^-3^, 3×10^-4^ ng/μl. Sterile water was used as a negative control. Three tubes of each concentration were set up for experiments. Do correlation regression analysis and determine the linear range according to the results.

#### 2.2.6 Double qPCR repeatability experiment

According to the sensitivity results, the concentration of DNA in the linear range was selected for repeatability experiment, and each tube of DNA was repeated three times, and the mean, standard deviation and coefficient of variation were calculated to evaluate the repeatability and stability of the method by calculating the Ct value of the three results.

#### 2.2.7 Clinical validation

Venous blood samples of patients with positive clinical blood culture were collected, The DNA of the bacteria was extracted from the blood samples with the QIAamp DNA purification mini kit, and then the double qPCR experiment was performed to determine whether the method could specifically detect CRAB in the patient’s BSIs.

## 3 Results

### 3.1 Dual qPCR system construction

**Figure 1:**
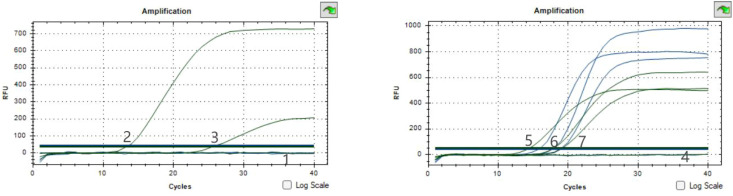
Results of constructing a dual qPCR system Note: The fluorescent color of 16sRNA is green; The fluorescent color of OXA-23 is Blue. 1 and 4 specimens are a negative control, 2 and 3 specimens are CSAB, 5-7 specimens are CRAB.

Using sterile water as the negative control, different CSABs and CRABs were selected for dual qPCR system construction, as shown in **Figure 1**. The fluorescence channel of the 16sRNA probe designed in this experiment was HEX(Excitation wavelength 515-535nm, emission wavelength 558nm,green), and the fluorescence channel of the OXA-23 probe was FAM(Excitation wavelength 450-490nm, emission wavelength 518nm, blue), CSAB only 16sRNA fluoresce, while CRAB both 16sRNA and OXA-23 fluoresce. The results of this experiment showed that the established dual qPCR system could detect CRAB.

### 3.2 Optimization of the dual qPCR reaction system

#### 3.2.1 Optimization of primer concentration in dual qPCR

**Figure 2:**
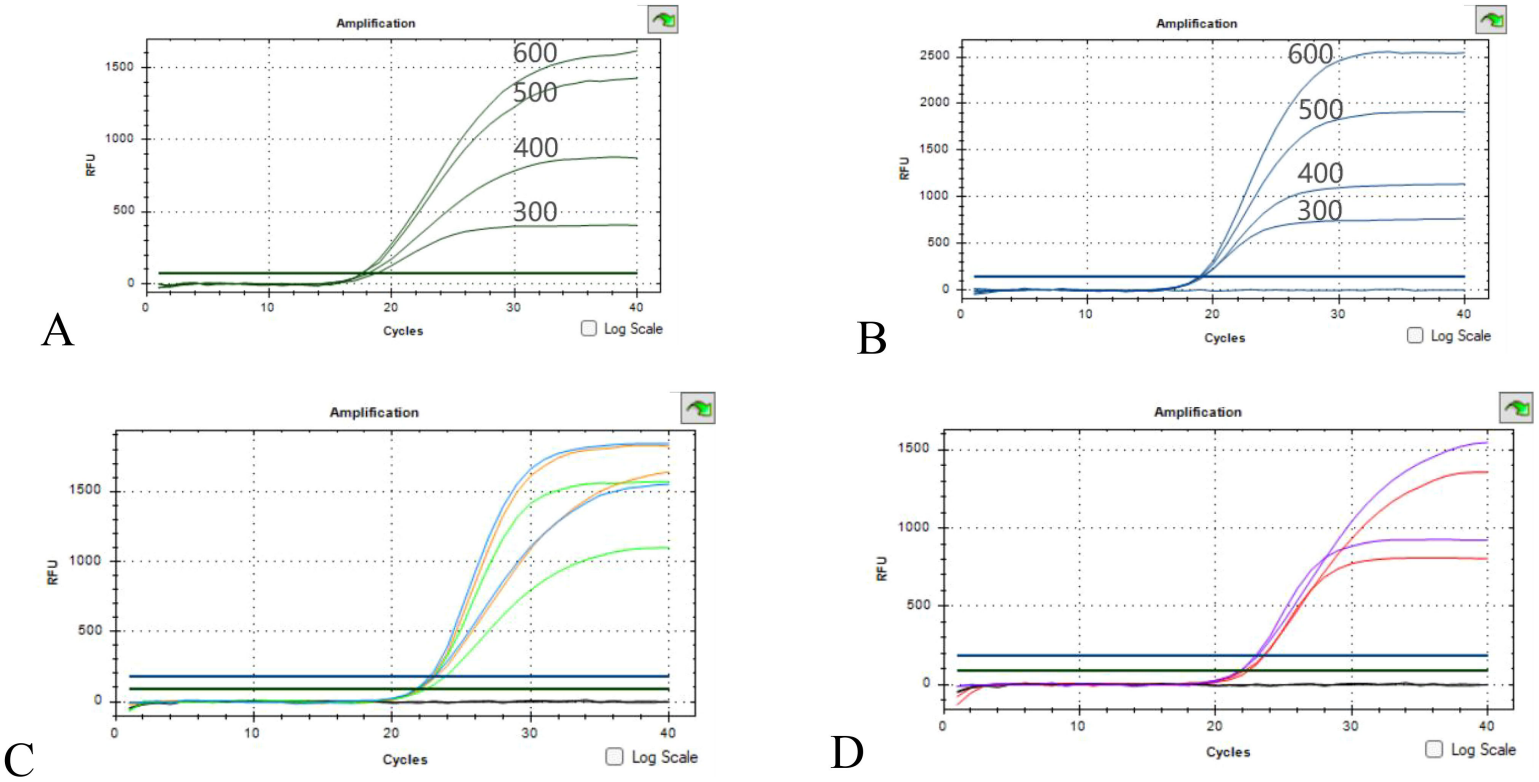
Optimization results of primer concentration and ratio **(A, B)** Optimization of 16sRNA/OXA-23 primers concentration; **(C)** Green concentration ratio of 500nM:400nM, orange concentration ratio of 500nM:500nM, blue concentration ratio of 600nM:500nM; **(D)** The red concentration ratio is 500 nM:300 nM, and the purple concentration ratio is 600 nM:400 nM.

In order to verify the effect of primer concentration on amplification, four primer concentrations of 300 nM, 400 nM, 500 nM and 600 nM were set up according to the common range of primer concentration, and the results are shown in **Figures 2A, B**. The Ct values of the four concentrations remained largely unchanged. The primer concentration had effect on the Ct value of 16sRNA and OXA-23 amplification, and the Ct value decreased with the increase of primer probe concentration and the fluorescence intensity. Considering that the fluorescence background should be minimized while increasing the sensitivity of the experiment, 500 nM was used as the optimal primer concentration for this experiment (**Supplementary Table S2**).

#### 3.2.2 Optimization of the dual qPCR 16sRNA/OXA-23 primer probe concentration ratio

16sRNA/OXA-23 primer concentrations are 500 nM:300 nM, 500 nM:400 nM, 500 nM:500 nM, 600 nM:500 nM, 600 nM:400 nM. As can be seen from the results of **Figures 2C, D**, there is no obvious promotion or inhibition relationship, and the 16sRNA/OXA-23 primer concentration ratio is selected as 500 nM:500 nM as the final primer concentration ratio in combination with Ct values and fluorescence intensity.

#### 3.2.3 Optimization of annealing temperature of dual qPCR

The results of annealing temperature optimization are shown in **Table 1**, when the annealing temperature decreases with 65°C, 64.5°C, 63.4°C, 61.9°C, 60°C, and 58.4°C, the Ct value decreases, and the fluorescence intensity increases. When it continues to decrease to 57.3°C or 56.7°C, the Ct value begins to rise and the fluorescence intensity also decreases. 16sRNA gave the best results at 58.4°C and OXA-23 at 58.4°C and 57.3°C. Therefore, 58.4°C was chosen as the annealing temperature for this experiment (**Supplementary Figure S1**).

**Table 1:** Optimization results of annealing temperature.

T (°C)	16sRNA Ct value	OXA-23 Ct value
65	21.45	22.64
64.5	21.23	22.32
63.4	21.02	22.17
61.9	21.29	22.23
60	21.11	22.16
58.4	20.79	21.95
57.3	21.05	21.94
56.7	21.05	22.16

### 3.3 Dual qPCR specificity validation

**Figure 3:**
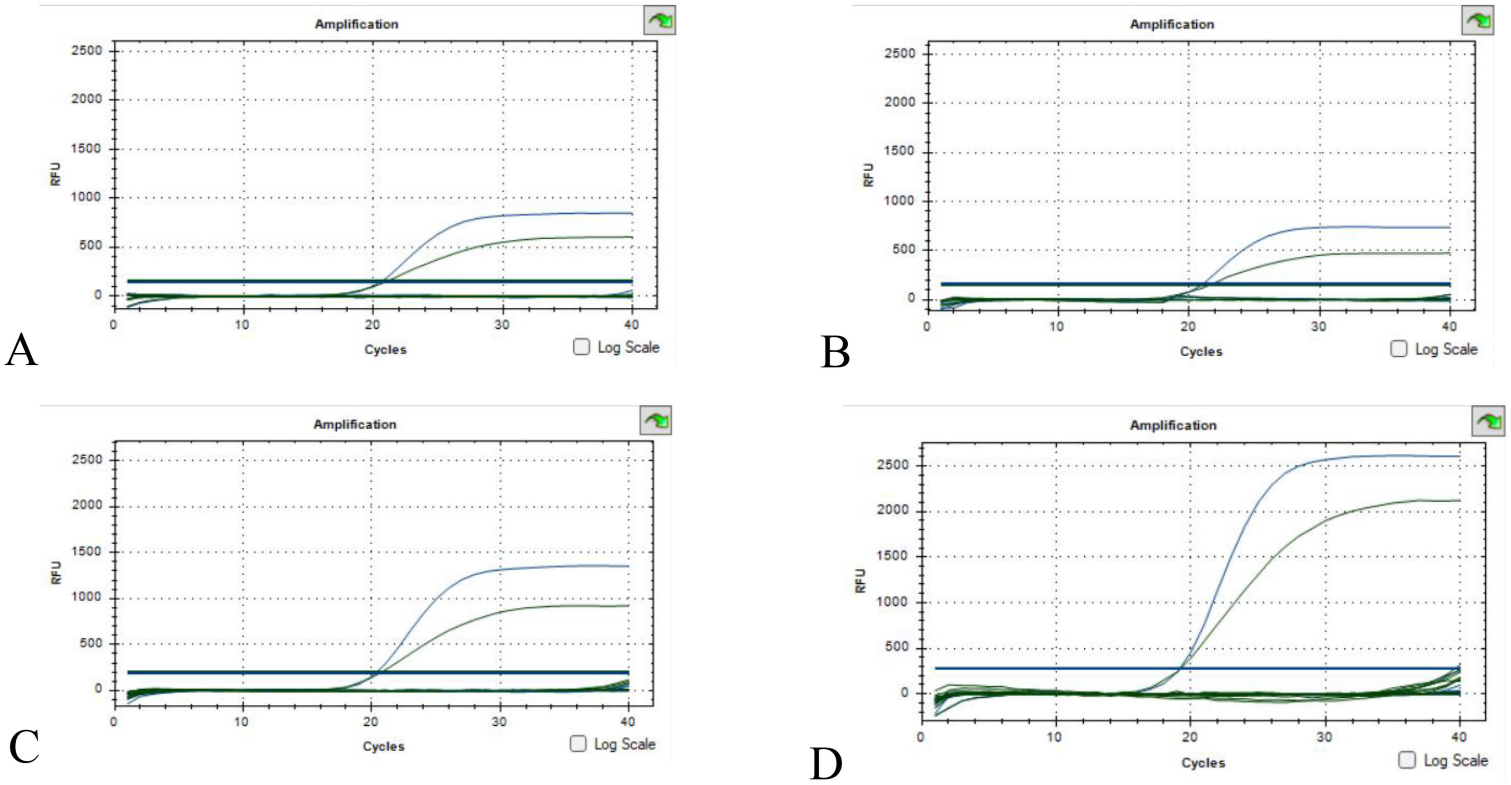
Specificity validation results **(A)** *Escherichia coli*, *Klebsiella pneumoniae*, *Staphylococcus epidermidis*, *Staphylococcus aureus*; **(B)** *Enterococcus faecalis*, *Staphylococcus hominis*, *Staphylococcus haemolyticus*, *Enterobacter cloacae*; **(C)** *Staphylococcus capitis*, *Pseudomonas aeruginosa*, *Enterococcus faecium*, *Streptococcus pneumoniae*, *Staphylococcus lugdunensis*, *Klebsiella oxytoca*, *Corynebacterium striatum*; **(D)** *Serratia marcescens*, *Staphylococcus warneri*, *Morganella morganii*, *Proteus mirabilis*, *Propionibacterium acnes*, *Stenotrophomonas maltophila*, *Hepatitis B* virus, *Candida albicans*, *Candida tropicalis*, *Candida glabrata*. (The green S-type curve being the 16sRNA positive control and the blue S-type curve being the OXA-23 positive control. Curves below the threshold line are negative).

The results of 22 bacteria, 3 fungi and 1 virus specificity are shown in **Figure 3**. A. baumanii was used as a positive control and sterile water was used as a negative control. The specificity verification results showed that there was no fluorescence in the number of non-A. baumanii, which was a negative result, indicating that the specificity of the methodology was good.

### 3.4 Dual qPCR detection limit and linearity experiment

**Figure 4:**
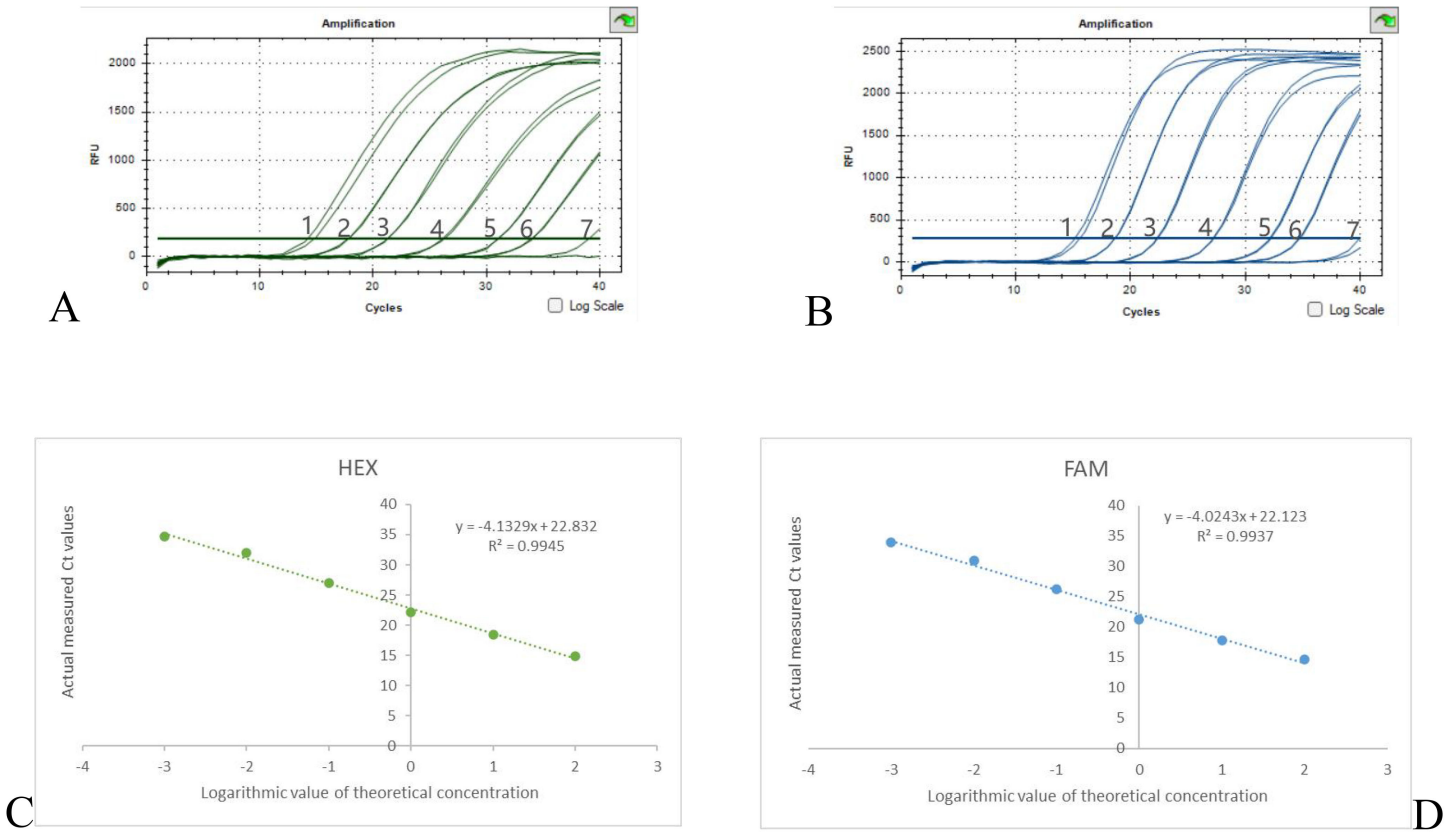
Results of sensitivity experiments and standard curves **(A)** 16sRNA sensitivity results, **(B)** OXA-23 sensitivity results, DNA concentration from 1-7 to 3×10^2^-3× 10^-4^ ng/μl. **(C)** 16sRNA standard curve; **(D)** OXA-23 standard curve.

The results of the dual qPCR sensitivity experiment are shown in **Figure 4**, when the DNA concentration is diluted to 3×10^-4^ ng/μl, there is no fluorescence production in both the 16sRNA and OXA-23 genes. Therefore, the minimum detection limit for both OXA-23 and 16sRNA detection by dual qPCR is 3×10^-3^ ng/μl. When A. baumanii DNA concentrations were in the range of 3×10^2^-3×10^-3^ ng/μl, the mean of the two Ct values for each concentration was calculated (**Supplementary Table S3**), and then the standard curves for 16sRNA and OXA-23 were y = -4.1329x + 22.832, R2 = 0.9945 and y = -4.0243x + 22.123, R2 = 0.9937 (**Figures 4C, D**), respectively. Therefore, the dual qPCR method had a good linear relationship between 16sRNA and OXA-23 and a wide linear range.

### 3.5 Dual qPCR repeatability experiment

According to the sensitivity and linearity experimental results, six concentrations of 3×10^2^-3×10^-3^ng/μl in the linear range were taken, and each concentration was repeated 3 times (**Supplementary Figure S2**). Calculate the mean of the Ct values and calculate the standard deviation and coefficient of variation(CV). From **Table 2**, it can be seen that the CV of each concentration is less than or equal to 2%, which has good repeatability.

**Table 2:** Repeatability experiment results.

Sample		Ct ( $\bar{\text{x}}$ )	SD	CV%
3×10^2^ng/µl	16sRNA	14.51	0.290	2.00
	OXA-23	15.23	0.305	2.00
3×10^1^ng/µl	16sRNA	17.82	0.050	0.28
	OXA-23	18.60	0.023	0.12
3×10^0^ng/µl	16sRNA	21.23	0.091	0.43
	OXA-23	22.29	0.060	0.27
3×10^-1^ng/µl	16sRNA	26.16	0.099	0.38
	OXA-23	27.16	0.072	0.27
3×10^-2^ng/µl	16sRNA	30.98	0.071	0.23
	OXA-23	32.14	0.057	0.18
3×10^-3^ng/µl	16sRNA	33.99	0.085	0.25
	OXA-23	34.82	0.232	0.67

### 3.6 Clinical validation

A total of 30 intravenous blood samples were collected on the same day after clinical blood culture **Table 3**. 9 negative blood culture samples, 6 blood culture positive but non-A. baumanii samples and 15 CSAB and CRAB samples. These specimens were tested for both qPCR (**Supplementary Figure S3**) and blood culture (susceptibility). The qPCR assay showed 100% sensitivity and specificity for the 30 whole blood sample(The criteria of positive is Ct<38).

**Table 3:** Clinical Validation of the qPCR assay.

qPCR BC	Number of bacterial	16sRNA	OXA23	Drug sensitivity results (phenotype)
negative	9	–	–	–
Staphylococcus aureus	2	–	–	Carbapenem Sensitive
Escherichia coli	2	–	–	Carbapenem Sensitive
Klebsiella pneumoniae	2	–	–	Carbapenem Sensitive
CSAB	4	+	–	Carbapenem Sensitive
CRAB	11	+	+	Carbapenem Resistance

## 4 Discussion

Currently, the gold standard for diagnosing BSIs remains a positive blood culture (BC). Relevant statistics point out that it takes about 1 to 5 days for the venous blood culture of clinical patients to be detected by the instrument, and then it takes 1 day for drug sensitivity after identification. Therefore, it takes at least two days from the collection of specimens to the final susceptibility report (Sinha et al., 2018). With data suggesting that an hour delay in antibiotic therapy is associated with an average increase in mortality of 7.6% in patients with sepsis, timely and accurate identification of the causative organism and selection of appropriate antimicrobial agents are critical for the treatment and prognosis of patients with early stages of BSIs (Zheng et al., 2021). Therefore, there is an urgent need to develop new technologies for the rapid diagnosis of BSIs.

Metagenomic next-generation sequencing (mNGS), droplet digital PCR (ddPCR), and fluorescence quantitative PCR(qPCR) are currently being studied for the diagnosis of BSIs (Peri et al., 2022). The amplification reaction of qPCR is usually completed within 2 hours, with an average cost of about $2-3 per person. The detection time of ddPCR is also relatively short, taking about 4-5 hours, but the system cost is relatively high($11-13); Due to the large amount of sequencing data, mNGS has a relatively long detection time, requiring at least 24 to 36 hours, and is relatively expensive (with a minimum cost of about $200) (Hu et al., 2021; Ngouth et al., 2022; Wang et al., 2022; Wissel et al., 2022; Bian et al., 2023; Zhang et al., 2023). mNGS and ddPCR are more time-consuming, more complex in operation and more expensive, which have limited their wide application in clinic (Kou et al., 2024). In addition, dual qPCR adds two pairs of primers to the same reaction and amplifies two gene fragments at the same time, which shows higher efficiency and lower cost (Sun et al., 2022). More importantly, qPCR has relatively good sensitivity and specificity for detecting microbial DNA (Tat Trung et al., 2018). But qPCR also has its limitations, such as being able to detect only specific known pathogens, susceptibility to inhibitors in clinical samples and lack of absolute quantification (Ma et al., 2020; Lima et al., 2022). At present, a certain number of qPCR technologies for the detection of bacteria have been applied in clinical practice, and their technology is relatively more mature and can be put into clinical application more quickly (Liu et al., 2018). To sum up, qPCR was used to establish the detection method of CRAB.

Recent studies of multiplex qPCR detection methods for BSIs have also demonstrated the feasibility of qPCR technology for the detection. The detection of *Staphylococcus aureus*, coagulase negative staphylococci, enterococci*, Enterobacteriaceae, Pseudomonas aeruginosa* and A. baumanii from BSIs was reported (Arabestani et al., 2014; Liu et al., 2018). However, there are few studies on the detection of CRAB. It is significance of determining carbapenem resistance in A. baumanii for the doctors. If it is CRAB, combination therapy is needed to achieve optimal results. For example, Meropenem in combination with baicalein exhibits synergism against extensively drug resistant and pan-drug-resistant A. baumanii *in vitro* (Güran et al., 2023).

Therefore, in order to overcome the problem, the method was established to detect CRAB by 16sRNA(A. baumanii) and OXA-23 genes, which could accurately distinguish A. baumanii from the most common pathogens in BSIs. The method has a high sensitivity (LOD: 3×10^-3^ng/μl); Wide linear range, positive results can be obtained in the concentration range of 3×10^2^-3×10^-3^ ng/μl, and the precision is good (CV ≤ 2%); This method can quickly detect CRAB in early stage, and the results are consistent with BC and drug susceptibility. So this method has a broad application prospect in the diagnosis of CRAB.

## Data Availability

The original contributions presented in the study are included in the article/**Supplementary Material**. Further inquiries can be directed to the corresponding authors.
