# Supplementary material for: Establishment and validation of a dual qPCR method for the detection of carbapenem-resistant Acinetobacter baumannii in bloodstream infections
Source: Front Cell Infect Microbiol. 2025 Feb 26;15:1490528. doi: 10.3389/fcimb.2025.1490528 (PMC11897477; doi:10.3389/fcimb.2025.1490528)
Supplement: Supplementary file 1 [file DataSheet1.docx]

**Supplementary material**

Table S1 Target gene primer sequences

| Genes | Sequence (5’→3’) |
| --- | --- |
| 16sRNA | F: AAG CGA GGA GGA GGC TAC TTT AG |
|  | R: CGG CTG CTG GCA CAG AGT |
|  | P: ACG TTA CTC GCA GAA TAA GCA CCG GCT |
| OXA-23 | F: GAC ACT AGG AGA AGC CAT GAA GCT |
|  | R: GCA TGA GAT CAA GAC CGA TAC G |
|  | P: TCC CAG TCT ATC AGG AAC TTG CGC G |

Table S2 Primer concentration ratio optimization results

| Concentration ratio | 16sRNA  Ct Value | OXA-23  Ct Value |
| --- | --- | --- |
| 500nm:300nm | 22.12 | 23.54 |
| 500nm:400nm | 22.43 | 23.05 |
| 500nm:500nm | 22.08 | 22.86 |
| 600nm:500nm | 21.75 | 22.68 |
| 600nm:400nm | 21.79 | 22.88 |

| A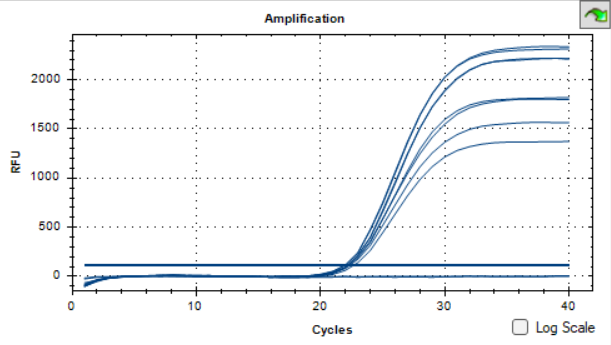 | B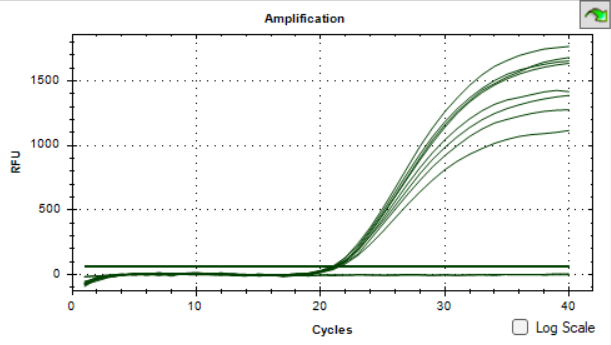 |
| --- | --- |

FigureS1 A 16sRNA annealing temperature optimization results; B OXA-23 annealing temperature optimization results

Table S3 Results of Ct values for sensitivity experiments

| Logarithmic value of DNA concentration | 16sRNA | OXA-23 |
| --- | --- | --- |
| -3 | 34.76 | 33.95 |
| -2 | 32.02 | 30.95 |
| -1 | 27.02 | 26.18 |
| 0 | 22.19 | 21.28 |
| 1 | 18.48 | 17.8 |
| 2 | 14.92 | 14.65 |

| A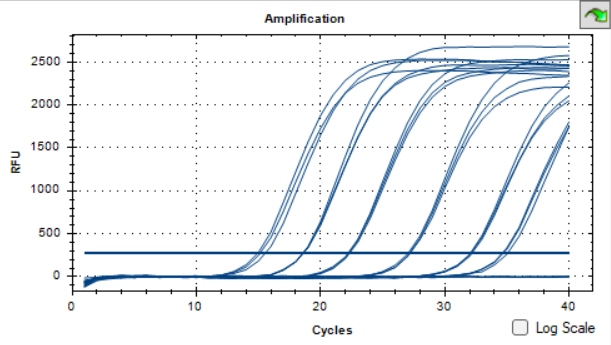 | B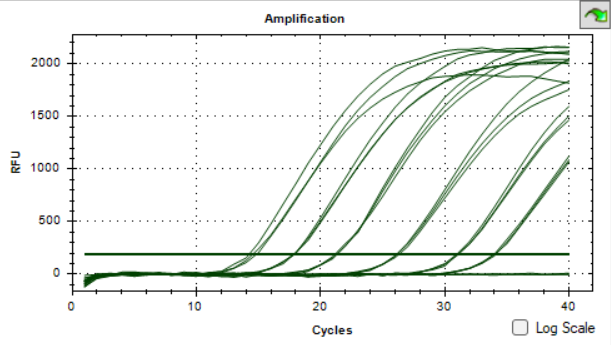 |
| --- | --- |

Figure S2 A: OXA-23 reproducibility experiment; B: 16sRNA reproducibility experiment

| A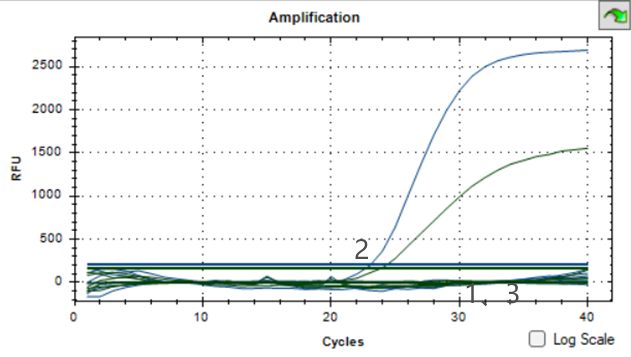 | |
| --- | --- |
| B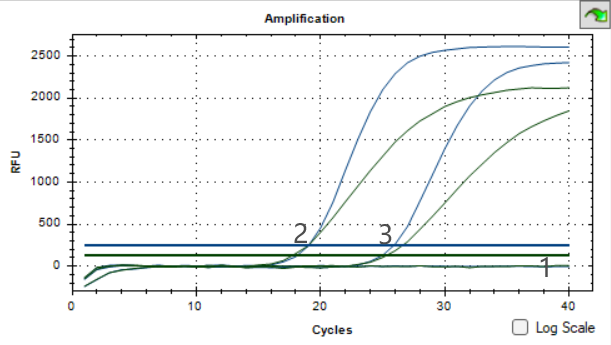 | C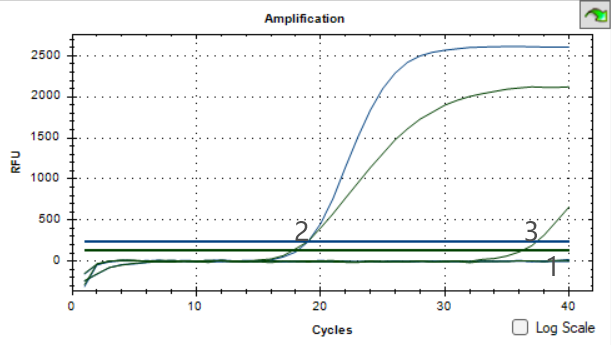 |
|  |  |

Figure S3. QPCR validation of clinical samples

A: negative specimen (curve below threshold is negative result.) , B: Blood specimen of CRAB; C: Blood specimen of CSAB

1: Negative Control, 2: positive control, 3: partial sample results (16sRNA in green, OXA-23 in blue) .
